# New Insights into LINC00346 and its Role in Disease

**DOI:** 10.3389/fcell.2021.819785

**Published:** 2022-01-13

**Authors:** Juan Lu, Zhaoying Xiao, Mengqiu Xu, Lanjuan Li

**Affiliations:** ^1^ State Key Laboratory for Diagnosis and Treatment of Infectious Diseases, National Clinical Research Center for Infectious Diseases, Collaborative Innovation Center for Diagnosis and Treatment of Infectious Diseases, The First Affiliated Hospital, College of Medicine, Zhejiang University, Hangzhou, China; ^2^ Department of Infectious Diseases Shengzhou People’ Hospital, Shengzhou Branch, The Fisrt Affiliated Hospital of Zhejiang University, Shengzhou, China

**Keywords:** lncRNA, LINC00346, biological function, mechanism, clinical utility

## Abstract

Accumulating evidence has shown that long intergenic non-protein-coding RNA 346 (LINC00346) functions as an oncogene in the tumorigenesis of several cancers. The expression level of LINC00346 has been shown to be obviously correlated with prognosis, lymphoma metastasis, histological grade, TNM stage, tumor size and pathologic stage. LINC00346 has been found to regulate specific cellular functions by interacting with several molecules and signaling pathways. In this review, we summarize recent evidence concerning the role of LINC00346 in the occurrence and development of diseases. We also discuss the potential clinical utility of LINC00346, thereby providing new insight into the diagnosis and treatment of diseases. In addition, we further discuss the potential clinical utility of LINC00346 in the diagnosis, prognostication, and treatment of diseases.

## Introduction

Human genome sequencing data have revealed that less than 2% of the human genome contains protein-coding genes, and the vast majority of genes give rise to noncoding RNAs (ncRNAs) (Li et al., 2018; He et al., 2019; Wei et al., 2019). ncRNAs, originally considered transcriptional noise (Yang X. et al., 2020; Wu et al., 2020), are considered essential regulators of gene expression; for example, they regulate transcription, mRNA stability, and mRNA translation (Fabian et al., 2010; Chen and Huang, 2018; Panni et al., 2020). There are multiple types of ncRNAs, such as microRNAs (miRNAs), small interfering RNAs (siRNAs), long noncoding RNAs (lncRNAs), and circular RNAs (Luo et al., 2017; Liu X. et al., 2019).

LncRNAs, typically longer than 200 nucleotides in length (Xu H. et al., 2020), lack an open reading frame of significant length (Liu X. et al., 2018; Li Y. et al., 2019; Parolia et al., 2019). Emerging evidence suggests that lncRNAs are key regulators of gene expression levels, posttranscriptional modifications, and binding to transcription factors or miRNAs (Zhao et al., 2019; Guo et al., 2021). LncRNAs are abnormally expressed in a plethora of diseases (Liu S. et al., 2019; Huang L. et al., 2019; Chen et al., 2019; Shen et al., 2019; Xing et al., 2019; Radhakrishnan and Kowluru, 2021). LncRNAs are correlated with a variety of clinical characteristics and are considered indispensable regulators of many cell activities, including cell proliferation, migration, invasion, and apoptosis (Yang et al., 2019; Li B. et al., 2021; Lei et al., 2021). LncRNAs play important roles in the occurrence and development of human diseases. These ideas provide new perspectives on the diagnosis and treatment of human diseases (Li L. et al., 2021; Ma et al., 2021; Morgan et al., 2021). Long intergenic non-protein-coding RNA 346 (LINC00346), a novel lncRNA, is encoded on chromosome 13q34. Several studies have revealed that LINC00346 is abnormally expressed in a variety of diseases and that aberrant LINC00346 expression is associated with many clinical features. LINC00346 has been found to regulate specific cellular functions by interacting with several molecular and signaling pathways. LINC00346 has also been identified as a potential biomarker in the diagnosis, prognostication, and treatment of diseases. In this review, we summarize current evidence concerning the expression, clinical characteristics, functions, and related mechanisms of LINC00346 in the occurrence and development of diseases. We also discuss the potential clinical utility of LINC00346, thereby providing new insight into the diagnosis and treatment of diseases.

## Role of LINC00346 in Disease

### Expression of LINC00346 in Disease

Increasing evidence has revealed that the expression level of LINC00346 is significantly upregulated in schizophrenia (Ghafouri-Fard et al., 2021), nasopharyngeal carcinoma (Cui et al., 2020), lung cancer (Wang et al., 2017), hepatocellular carcinoma (HCC) (Jin et al., 2020; Yin et al., 2020; Zhang and Chen, 2020), glioma (Yang C. et al., 2020; Chen X. et al., 2020), colorectal cancer (CRC) (Tong et al., 2020), cutaneous squamous cell carcinoma (Piipponen et al., 2020), breast cancer (Li et al., 2020d), gastric cancer, and pancreatic cancer (Table 1). LINC00346 may have a pathogenic role in disease progression. Interestingly, Gheliji et al. (2020) found that LINC00346 expression was decreased in lung cancer tissues compared with adjacent normal tissues. The expression level of LINC00346 needs to be further explored in lung cancer.

**TABLE 1:** The expression and clinical features of LINC00346 in disease.

Type	Expression	Feature	Refs
Epilepsy	—	Vitamin D level	Mazdeh et al. (2019)
Schizophrenia	Upregulated	Sex difference	Ghafouri-Fard et al. (2021)
Gastric cancer	Upregulated	Tumor size, pathologic stage, and disease-free survival	Xu et al. (2019)
Colorectal cancer	—	Lymphoma metastasis, histological grade, and TNM stage	Li et al. (2020b)
Hepatocellular carcinoma	Upregulated	—	Jin et al. (2020)
Hepatocellular carcinoma	Upregulated	—	Zhang and Chen, (2020)
Hepatocellular carcinoma	Upregulated	—	Yin et al. (2020)
Pancreatic cancer	Upregulated	Disease-free survival and overall survival	Peng et al. (2019)
Pancreatic cancer	Upregulated	—	Shi et al. (2019)
Pancreatic cancer	Upregulated	Overall survival	Zhang et al. (2018)
Glioma	—	Overall survival	Geng et al. (2020)
Glioma	Upregulated	Disease-free survival and overall survival	Chen et al. (2020b)
Glioma	Upregulated	—	Yang et al. (2020a)
Lung cancer	Upregulated	Sex difference	Gheliji et al. (2020)
Lung cancer	Upregulated	—	Wang et al. (2017)
Lung cancer	—	Overall survival	Wang et al. (2021c)
Breast cancer	Upregulated	—	Li et al. (2020d)
Breast cancer	—	Overall survival	Liu et al. (2016)
Nasopharyngeal carcinoma	Upregulated	Overall survival and recurrence-free survival	Cui et al. (2020)
Cutaneous squamous cell carcinoma	Upregulated	—	Piipponen et al. (2020)

### LINC00346 and Clinical Characteristics

Some research groups have reported a potential relationship between LINC00346 expression and clinicopathological features (Table 1). The expression of LINC00346 has the potential to indicate the prognosis of numerous diseases, such as nasopharyngeal carcinoma (Cui et al., 2020), lung cancer (Wang et al., 2017), glioma (Yang C. et al., 2020; Chen X. et al., 2020), breast cancer (Li et al., 2020d), and pancreatic cancer (Zhang et al., 2018; Peng et al., 2019; Shi et al., 2019). In addition, LINC00346 expression was found to be strongly correlated with metastasis, histological grade, and TNM stage in (Li T. et al., 2020) CRC. An increased LINC00346 level predicted larger tumor size and poorer pathologic stage in gastric cancer (Xu et al., 2019). The level of LINC00346 was relatively correlated with sex in lung cancer and schizophrenia patients (Gheliji et al., 2020; Ghafouri-Fard et al., 2021). The LINC00346 expression level was negatively associated with the vitamin D level in epileptic patients (Mazdeh et al., 2019).

### Functional Roles of LINC00346 in Disease

A growing amount of evidence has shown that lncRNAs play an important role in human disease (Zheng et al., 2019; Yu et al., 2020; Luo et al., 2021). LINC00346 exerts a vital role in the development of the disease by regulating various cellular functions. The regulatory functions of LINC00346 are related to cell proliferation, migration, invasion, and apoptosis. In this section, we summarize the current findings on the functions of LINC00346 (Table 2).

**TABLE 2:** The functions and mechanisms of LINC00346 in disease.

Type	Role	Function	Related genes	Refs
Schizophrenia	Oncogene	—	—	Ghafouri-Fard et al. (2021)
Atherosclerosis	—	Inflammatory factors and functional injury	miR-148a-3p, HUVECs, and KLF5	Wang et al. (2021a)
Gastric cancer	Oncogene	Cell proliferation, cell migration, cell invasion, and cell cycle	KLF5, MYC and miR-34a-5p	Xu et al. (2019)
Colorectal cancer	—	Cell proliferation, apoptosis, cell migration, and cell invasion	miR-148b	Li et al. (2020b)
Colorectal cancer	—	Cancer stemness properties	miR-509-5p and WBSCR22	Zhao et al. (2020)
Hepatocellular carcinoma	Oncogene	Apoptosis, cell migration, and cell cycle	miR-199a-3p, CDK1, CCNB1, and p53	Jin et al. (2020)
Hepatocellular carcinoma	Oncogene	Cell proliferation, cell migration, cell invasion	miR-542-3p, WDR18, Wnt/β-catenin pathway, and MYC	Zhang and Chen, (2020)
Hepatocellular carcinoma	Oncogene	Cell proliferation, apoptosis, cell migration, and cell invasion	JAK and STAT3	Yin et al. (2020)
Pancreatic cancer	Oncogene	Cell proliferation, cell migration, cell invasion	C-Myc and CTCF	Peng et al. (2019)
Pancreatic cancer	Oncogene	Cell proliferation, cell cycle, chemoresistance	miR-188-3p and BRD4	Shi et al. (2019)
Pancreatic cancer	Oncogene	Cell proliferation	—	Zhang et al. (2018)
Glioma	—	—	miR-128-3p and SZRD1	Geng et al. (2020)
Glioma	oncogene	Cell proliferation, apoptosis, cell migration, and cell invasion	miR-340-5p and ROCK1	Chen et al. (2020b)
Glioma	Oncogene	Angiogenesis	ANKHD1, LINC00346, and ZNF655	Yang et al. (2020a)
Lung cancer	Tumor suppressor	—	—	Gheliji et al. (2020)
Lung cancer	Oncogene	Cell proliferation, apoptosis, and cell cycle	JAK and STAT3	Wang et al. (2017)
Lung cancer	—	Cell proliferation, apoptosis, cell migration, cell invasion, and cell cycle	miR-30c-2-3 and MYBL2	Xu et al. (2021)
Lung cancer	—	Chemoresistance	—	Wang et al. (2021c)
Breast cancer	Oncogene	Cell proliferation, apoptosis, and glycolysis	miR-148a/b and GLUT1	Li et al. (2020d)
Breast cancer	—	—	—	Liu et al. (2016)
Bladder cancer	—	Cell proliferation, apoptosis, cell migration, and cell cycle	—	Ye et al. (2017)
Nasopharyngeal carcinoma	Oncogene	Chemoresistance	miR-342-5p	Cui et al. (2020)
Cutaneous squamous cell carcinoma	Oncogene	Cell invasion	STAT3 and MMP	Piipponen et al. (2020)

#### The Role of LINC00346 in Cellular Growth

Malignant diseases are often caused by unregulated cell growth (Huang et al., 2016; Emmanuel et al., 2018; Tan et al., 2019). Controlling cell growth is critical for the treatment of some diseases. The upregulation of LINC00346 has been found to promote cell proliferation and inhibit cell apoptosis in many diseases. Silencing LINC00346 has been found to obviously inhibit cell proliferation and promote cell apoptosis in bladder cancer (Ye et al., 2017), lung cancer (Wang et al., 2017; Xu et al., 2021), (Jin et al., 2020; Yin et al., 2020) HCC, glioma (Chen X. et al., 2020), (Li T. et al., 2020) CRC, and breast cancer (Li et al., 2020d). Additionally, several studies have revealed that elevated LINC00346 expression enhances cell proliferation in gastric cancer (Xu et al., 2019) and pancreatic cancer (Zhang et al., 2018; Peng et al., 2019; Shi et al., 2019). Cell cycle arrest induced by LINC00346 has been observed in bladder cancer (Ye et al., 2017), lung cancer (Wang et al., 2017; Xu et al., 2021), (Jin et al., 2020) HCC, gastric cancer (Xu et al., 2019), and pancreatic cancer (Shi et al., 2019). Knockdown of LINC00346 facilitates G1/G0 cell cycle arrest in bladder cancer (Ye et al., 2017), non-small-cell lung cancer (Wang et al., 2017; Xu et al., 2021), and (Jin et al., 2020)HCC. In gastric cancer, increased LINC00346 levels suppress cell cycle arrest at the G1–S phase (Xu et al., 2019) transition. The cell cycle is blocked in the G2/M phase with upregulated LINC00346 expression in pancreatic cancer (Shi et al., 2019) and lung adenocarcinoma (Xu et al., 2021).

#### The Role of LINC00346 in Cell Motility

Metastasis to adjacent and distant sites, indicating poor prognosis, is a sign of malignant disease (Chakraborty et al., 2008; Okugawa et al., 2014; Lin et al., 2020). Cell motility is a highly regulated mechanical process important in wound healing, metastasis, and embryogenesis (Selmeczi et al., 2005; Yip et al., 2007; Mejean et al., 2009). Increased expression of LINC00346 has been found to promote tumor cell metastasis in bladder cancer (Ye et al., 2017), lung cancer (Xu et al., 2021), (Jin et al., 2020; Yin et al., 2020; Zhang and Chen, 2020) HCC, glioma (Chen X. et al., 2020), (Li T. et al., 2020; Zhao et al., 2020) CRC, cutaneous squamous cell carcinoma (Piipponen et al., 2020), gastric cancer (Xu et al., 2019), and pancreatic cancer (Peng et al., 2019). LINC00346 significantly enhances cell migration and invasion in lung adenocarcinoma (Xu et al., 2021), (Jin et al., 2020; Yin et al., 2020; Zhang and Chen, 2020) HCC, glioma (Chen X. et al., 2020), (Li T. et al., 2020; Zhao et al., 2020)CRC, gastric cancer (Xu et al., 2019), and pancreatic cancer (Peng et al., 2019). LINC00346 also promotes cell migration in bladder cancer (Ye et al., 2017) and cell invasion in cutaneous squamous cell carcinoma (Piipponen et al., 2020).

#### The Role of LINC00346 in Drug Resistance

The increasing frequency of DR has prompted substantial research interest (Shehata, 2005; Shi and Gao, 2016; Carné Trécesson et al., 2017). DR remains a significant obstacle in the treatment of cancer. It is important to investigate the underlying mechanisms of chemoresistance for cancer treatment. The expression of LINC00346 was found to be markedly correlated with DR in nasopharyngeal carcinoma (Cui et al., 2020) and pancreatic cancer (Shi et al., 2019). Knockdown of LINC00346 enhanced the sensitivity to cisplatin in nasopharyngeal carcinoma cell lines (Cui et al., 2020). Silencing LINC00346 attenuated the gemcitabine tolerance in pancreatic cancer cell lines (Shi et al., 2019). Monitoring and modulating LINC00346 expression may be potential therapeutic strategies for guiding clinical treatment.

#### The Role of LINC00346 in Other Functions

Inflammation is considered a key factor in the pathophysiology of atherosclerosis (Sivapalaratnam et al., 2011; Paramel et al., 2020; Good et al., 2021). The upregulation of LINC00346 promoted inflammatory factor expression and functional injury in human umbilical vein endothelial cells (HUVECs) stimulated by OX-LDL (Wang F. et al., 2021). LINC00346 facilitated angiogenesis of glioma-associated endothelial cells (GECs), and *in vitro* LINC00346 knockdown experiments further verified this result (Yang C. et al., 2020). Glycolysis is a vital feature of tumor cells (Wang et al., 2013; Deng et al., 2020; Zhang et al., 2021). Inhibition of glycolysis is a promising therapeutic strategy for inhibiting tumors (Lee et al., 2017; Du et al., 2020). Increased LINC00346 levels were found to significantly promote glycolysis ability in breast cancer cell lines (Li et al., 2020d). In CRC, LINC00346 was found to regulate cancer stemness properties *in vitro* (Zhao et al., 2020).

### LINC00346 Regulatory Mechanisms in Disease

#### Mechanisms of LINC00346 in Tumors

##### Mechanisms of LINC00346 in Digestive System Tumors

**FIGURE 1:**
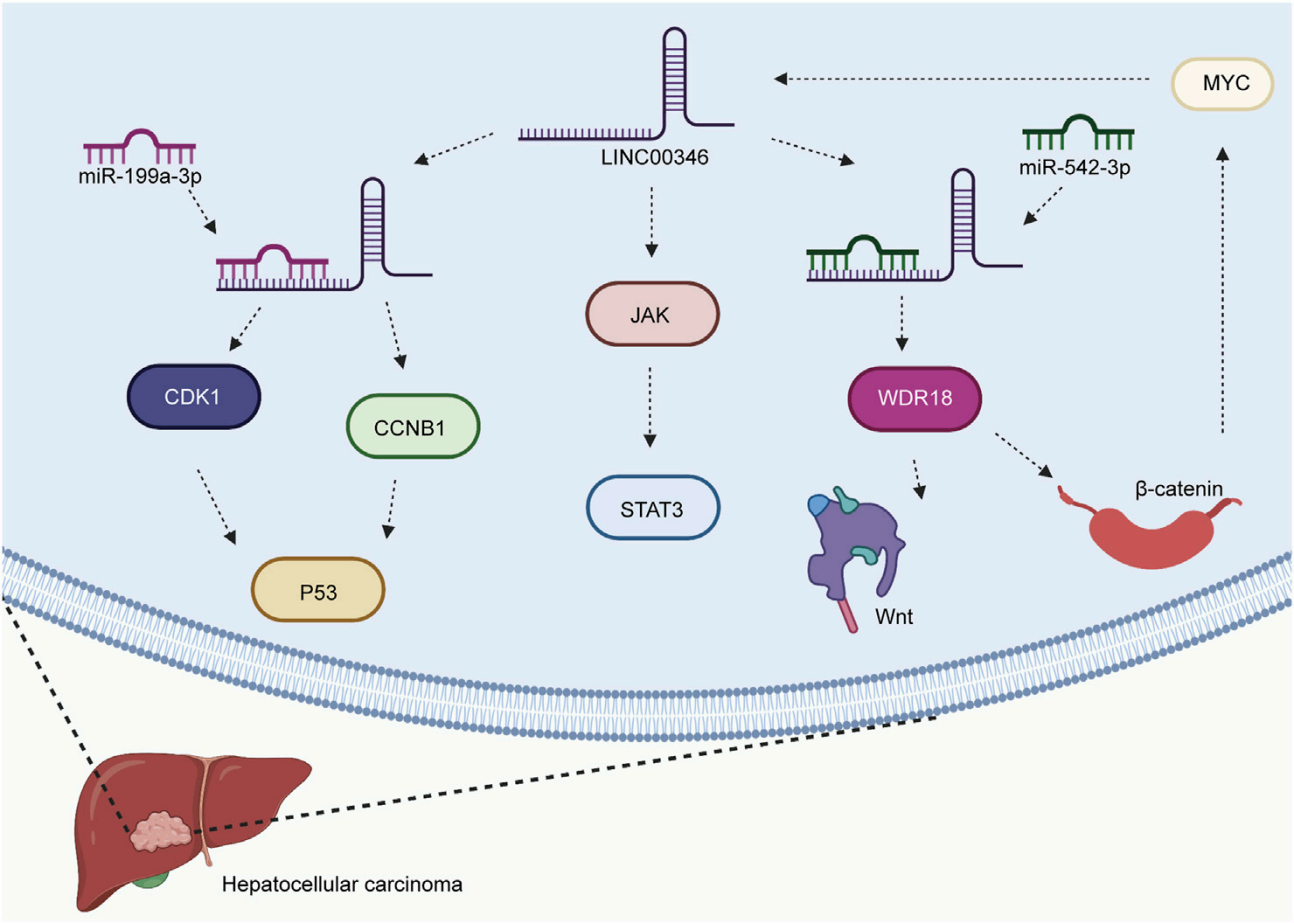
Mechanisms of LINC00346 in HCC (HCC). LINC00346 upregulates the expression levels of CDK1 and CCNB1 by sponging miR-199a-3p in HCC. It attenuates cell invasion and apoptosis and to regulates the cell cycle by regulating p53 and the miR-199a-3p/CDK1/CCNB1 axis. LINC00346 facilitates WDR18 expression and activates the Wnt/β-catenin pathway by acting as a sponge of miR-542-3p in HCC. β-catenin and LINC00346 form a positive feedback loop by interacting with MYC. Finally, LINC00346 affects cell proliferation and survival by activating the JAK-STAT3 signaling pathway.

HCC is the most common type of liver cancer and has increasing mortality worldwide (Gomha et al., 2015; Shen et al., 2018; Yapasert et al., 2020). The underlying signaling mechanisms of HCC progression are poorly defined. A growing amount of evidence has shown that LINC00346 is significantly associated with the progression of HCC. Upregulation of LINC00346 was found to promote cancer progression by regulating various biological functions in HCC. LINC00346 promoted the expression levels of CDK1 and CCNB1 by acting as a sponge of miR-199a-3p in HCC (Jin et al., 2020) (Figure 1). In addition, LINC00346 inhibited cell invasion and apoptosis and controlled the cell cycle by regulating p53 and the miR-199a-3p/CDK1/CCNB1 axis. LINC00346 facilitated WDR18 expression and the Wnt/β-catenin pathway by sponging miR-542-3p in HCC (Zhang and Chen, 2020). Researchers also observed that β-catenin and LINC00346 form a positive feedback loop by interacting with MYC. LINC00346 was also found to affect cell proliferation and survival by regulating the JAK-STAT3 signaling pathway (Yin et al., 2020).

Pancreatic carcinoma is one of the most malignant tumors and has an extremely poor prognosis (Xu D. et al., 2020; Wang et al., 2020; Chen et al., 2021). Chemotherapy is an important method of adjuvant therapy in the comprehensive treatment of pancreatic cancer (Rahman et al., 2017; Zhu et al., 2019). Therefore, the chemoresistance and pathogenesis of pancreatic cancer urgently need to be explored. LINC00346 was found to facilitate the transcription and expression of c-Myc by interacting with CTCF in pancreatic cancer (Peng et al., 2019). LINC00346, acts as a sponge of miR-188-3p and downregulated the level of BRD4 to increase gemcitabine resistance in pancreatic cancer (Shi et al., 2019). In CRC, LINC00346 promotes cell migration and invasion by reducing miR-148b levels (Li T. et al., 2020). LINC00346 also regulates the biological functions of CRC stem cells by activating the Linc00346/miR-509-5p/wbscr22 pathway (Zhao et al., 2020). LINC00346 suppresses miR-34a-5p expression to affect CD44, Notch1, and AXL expression in gastric cancer (Xu et al., 2019). In addition, the expression of LINC00346 was found to be markedly upregulated by KLF5 and MYC in gastric cancer.

##### Mechanisms of LINC00346 in Central Nervous System Tumors

Gliomas are the most common type of tumor of the central nervous system (Ceresa et al., 2019; Liu Y. et al., 2019; Stępniak et al., 2021).

**FIGURE 2:**
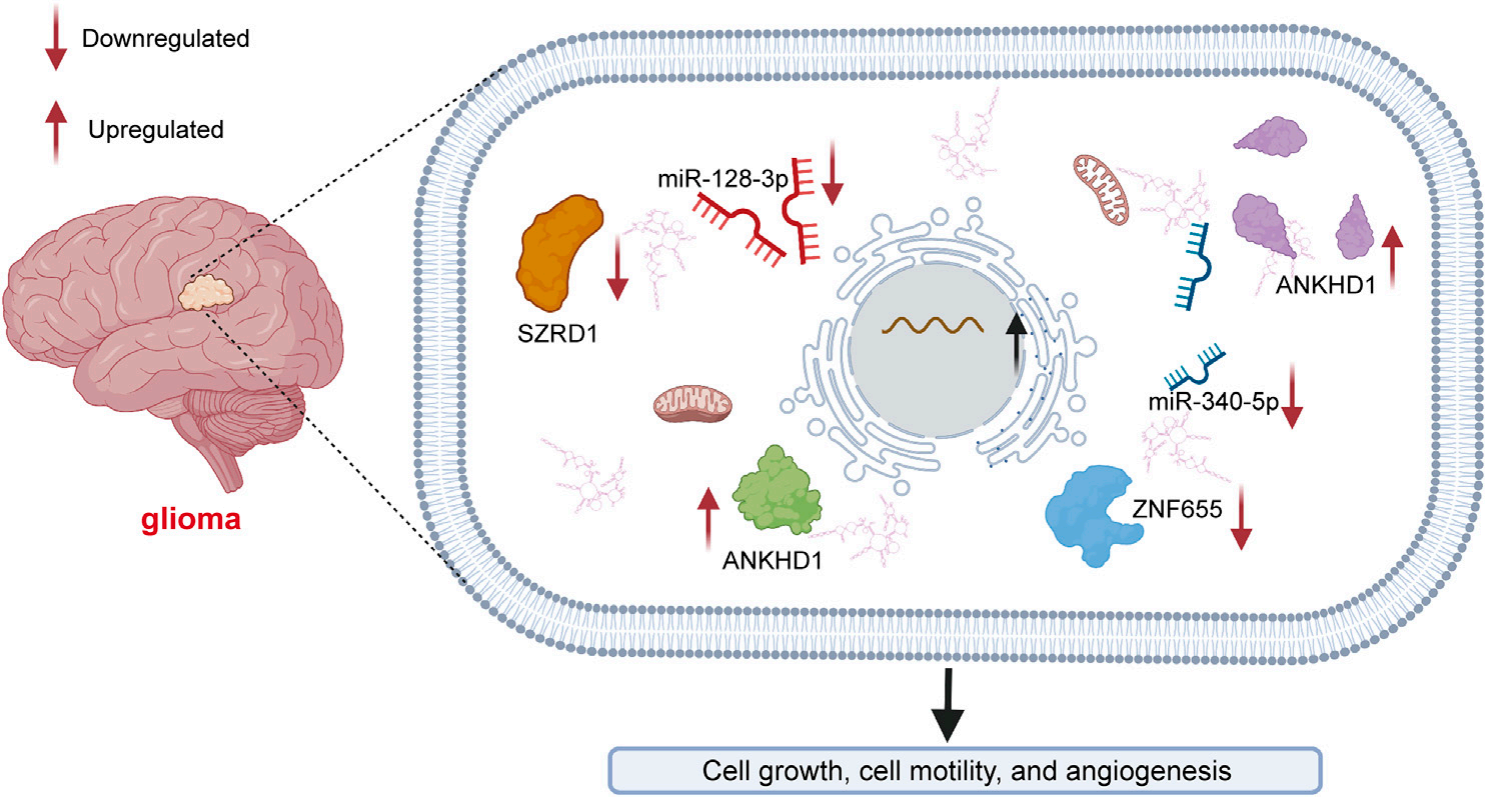
Mechanisms of LINC00346 in glioma. LINC00346 upregulates SZRD1 levels by sponging miR-128-3p in glioma. LINC00346 modulates cell proliferation and apoptosis by regulating miR-128-3p/SZRD1. LINC00346 also functions as a ceRNA of miR-340-5p to reduce the expression level of ROCK1 in glioma. The expression level of LINC00346 is positively regulated by ankyrin repeat and KH domain-containing protein 1 (ANKHD1). Angiogenesis associated with glioma-associated endothelial cells (GECs) is facilitated by the ANKHD1/LINC00346/znf655 pathway.

Gliomas are the most aggressive type of brain tumor and have an extremely poor prognosis (Seliger and Hau, 2018; Chen W. et al., 2020). Glioma can be divided into astrocytoma, glioblastoma multiforme (GBM), oligodendroglioma and mixed tumors (Peng et al., 2018). Geng et al. (2020) found that LINC00346 inhibited the expression of miR-128-3p to upregulate SZRD1 levels in glioma (Figure 2). LINC00346 was found to affect cell proliferation and apoptosis through the regulation of miR-128-3p/SZRD1. LINC00346 was also found to act as a ceRNA (competing endogenous RNA) of miR-340-5p to suppress the expression of ROCK1 in glioma progression (Chen X. et al., 2020). Exploring the potential angiogenesis mechanisms is essential for the development of novel strategies for glioma treatment. LncRNAs play an essential role in tumor angiogenesis (Ruan et al., 2019; Cheng et al., 2020; Wang X. et al., 2021). In glioma, the expression level of LINC00346 was found to be positively regulated by ankyrin repeat and KH domain-containing protein 1 (Yang C. et al., 2020) (ANKHD1). The activation of ANKHD1/LINC00346/znf655 was found to facilitate angiogenesis in association with glioma-associated endothelial cells (GECs).

##### Mechanisms of LINC00346 in Tumors of Other Systems

Lung cancer is the leading cause of cancer-related deaths worldwide (Liu J. et al., 2018; Harikrishnan et al., 2020; To et al., 2021). It can be divided into small-cell lung cancer and non-small-cell lung cancer (NSCLC) (Du et al., 2019; Li H. et al., 2020). LINC00346 was found to facilitate NSCLC progression via regulation of the JAK-STAT3 signaling pathway (Wang et al., 2017). LINC00346 promotes the expression of MYBL2 to regulate the cell cycle by acting as a sponge of miR-30c-2-3p in lung adenocarcinoma (Xu et al., 2021). Nasopharyngeal carcinoma (NPC) is a head and neck malignancy with a high incidence (Sun and Xu, 2015; Yao et al., 2020). Chemoresistance remains an obstacle in the treatment of NPC. LINC00346 attenuates cisplatin sensitivity by sponging miR-342-5p in NPC (Cui et al., 2020). In breast cancer, the expression of LINC00346 upregulates glucose transporter 1 levels by targeting miR-148a/b (Li et al., 2020d). Cutaneous squamous cell carcinoma is the second most frequent malignant skin cancer, and the incidence is increasing. LINC00346 increases the expression of matrix metalloproteinase by activating STAT3 signaling in cutaneous squamous cell carcinoma (Piipponen et al., 2020).

#### Mechanisms of LINC00346 in Nontumor Disease

Atherosclerosis is a major cause of multiple diseases, such as coronary artery disease (CHD) (Martinus and Goldsbury, 2018; Xu et al., 2018), peripheral artery disease (PAD) (Biscetti et al., 2019), and atherosclerotic cerebrovascular disease (Sascău et al., 2021). However, the pathogenesis of atherosclerosis remains unclear. There is an urgent need to explore the mechanism of atherosclerosis. The level of LINC00346 was found to be negatively correlated with Krüppel-like factor 5 (KLF5) expression in atherosclerosis (Wang F. et al., 2021). Knockdown of LINC00346 inhibited inflammatory reactions and functional injury in the progression of atherosclerosis. LINC00346 was found to affect the initiation and development of atherosclerosis by regulating the KLF5/LINC00346/miR-148a-3p pathway (Wang F. et al., 2021).

### Clinical Utility of LINC00346 in Disease

Despite improved technology and advances in modern medicine, malignant disease, especially cancer, remains one of the leading causes of death. Abnormal cell growth, metastasis, and drug assistance result in poor disease prognoses. Early diagnosis and targeted treatment are important for improving the prognosis of disease. LncRNAs may be potential diagnostic biomarkers and therapeutic targets. In this section, we will further discuss the potential clinical utility of LINC00346 in the diagnosis, prognostication, and treatment of diseases.

#### LINC00346 as a Diagnostic Biomarker

The detection and diagnosis of disease are essential for disease management (Li et al., 2020c; Álvarez et al., 2020; Adhikari et al., 2021). Increasing evidence has revealed that lncRNAs are potential diagnostic biomarkers for several diseases (Wang et al., 2016; Jin et al., 2017; Huang G.-z. et al., 2019; Cheng et al., 2019). LINC00346 has found to be obviously upregulated in many tumors and nontumor diseases. Gheliji et al. (2020) observed that LINC00346 expression was downregulated in lung cancer tissues. The level of LINC00346 in lung cancer needs to be further explored. The sensitivity was 83.3%, and the specificity was 52.4% in an analysis of the ability to distinguish between lung cancer tissues and adjacent tissues (Gheliji et al., 2020). Importantly, the expression of LINC00346 in venous blood was found to be markedly upregulated in patients with schizophrenia or pancreatic cancer (Zhang et al., 2018; Ghafouri-Fard et al., 2021). LINC00346 has great value in the diagnosis of schizophrenia (Ghafouri-Fard et al., 2021) and pancreatic cancer (Zhang et al., 2018). Substances that are stably expressed in body fluids are more likely to be used as biomarkers in disease diagnosis.

#### LINC00346 as a Prognostic Biomarker

Individualized therapy requires the identification of biomarkers to predict patient prognosis (Li Z. et al., 2019; Melling et al., 2019). The expression of LINC00346 was found to be significantly correlated with the poor prognosis of nasopharyngeal carcinoma (Cui et al., 2020), lung cancer (Wang et al., 2017), glioma (Yang C. et al., 2020; Chen X. et al., 2020), breast cancer (Li et al., 2020d), and pancreatic cancer (Zhang et al., 2018; Peng et al., 2019; Shi et al., 2019). The level of LINC00346 was found to be negatively associated with overall survival in nasopharyngeal carcinoma (Cui et al., 2020), lung adenocarcinoma (Wang Z. et al., 2021), glioma (Chen X. et al., 2020; Geng et al., 2020), breast cancer (Liu et al., 2016), and pancreatic cancer (Zhang et al., 2018; Peng et al., 2019). LINC00346 was also found to affect disease-free survival in patients with gastric cancer (Xu et al., 2019) and pancreatic cancer (Peng et al., 2019). The upregulation of LINC00346 was found to be correlated with shorter recurrence-free survival in nasopharyngeal carcinoma (Cui et al., 2020).

#### LINC00346 as a Biomarker of Targeted Therapy

Molecular targeted therapy shows advantages for many diseases, especially malignancies (Baba et al., 2019; Becker et al., 2020; Xu P. et al., 2020). LncRNAs contribute to disease progression through the regulation of cellular pathways (Li Z.-W. et al., 2020; Sun and Wu, 2020). They serve as an important therapeutic targets in the treatment of diseases. In Section 2.4, we introduced the mechanisms of LINC00346 in tumor and nontumor diseases. LINC00346 is a meaningful therapeutic biomarker in disease treatment. It is also considered an oncogene that contributes to tumorigenesis. Knockdown or silencing of LINC00346 can inhibit cell biological functions to suppress cancer progression in several cancers, such as bladder cancer (Ye et al., 2017), lung cancer (Wang et al., 2017), and glioma (Chen X. et al., 2020; Geng et al., 2020). The association of LINC00346 and chemoresistance has implications for treatment.

## Conclusions and Future Perspectives

LINC00346, a novel lncRNA, is encoded on chromosome 13q34. It is significantly upregulated in many diseases. However, some researchers have found that the level of LINC00346 is reduced in lung cancer. Therefore, the expression of LINC00346 in lung cancer needs to be further explored. Importantly, the expression of LINC00346 in venous blood was found to be elevated in patients with schizophrenia or pancreatic cancer. This finding is crucial for the successful clinical application of LINC00346. Substances stably expressed in bodily fluids have strong potential in the diagnosis of disease. The expression level of LINC00346 was found to be obviously correlated with prognosis, lymphoma metastasis, histological grade, TNM stage, tumor size and pathologic stage. LINC00346 expression has important guiding significance in the management of patients. Different strategies can be used for different patients if the prognosis can be accurately predicted. LINC00346 exerts a vital role by regulating cellular growth, cell motility, chemoresistance, and other functions in diseases. LINC00346 affects these biological functions by interacting with several pathways. Knockdown or silencing of LINC00346 inhibits the progression of several cancers. In conclusion, LINC00346 is a potential biomarker in the diagnosis, prognostication, and treatment of diseases. In terms of clinical applications, further basic experiments and multicenter research data are needed.
